# Levels of actigraphy-derived physical activity among Polish nurses: factors associated with the prevalence of selected metabolic disorders

**DOI:** 10.3389/fpubh.2023.1300662

**Published:** 2023-12-29

**Authors:** Anna Bartosiewicz, Piotr Matłosz, Justyna Wyszyńska, Edyta Łuszczki, Łukasz Oleksy, Olga Adamska, Alejandro Martínez-Rodríguez, Artur Mazur

**Affiliations:** ^1^Institute of Health Sciences, Medical College of Rzeszów University, Rzeszów, Poland; ^2^Institute of Physical Culture Sciences, Medical College of Rzeszów University, Rzeszów, Poland; ^3^Department of Physiotherapy, Faculty of Health Sciences, Jagiellonian University Medical College, Kraków, Poland; ^4^Department of Orthopedics and Rehabilitation, Medical University of Warsaw, Warsaw, Poland; ^5^Department of Analytical Chemistry, Nutrition and Food Science, University of Alicante, Alicante, Spain; ^6^Institute of Medical Sciences, Medical College of Rzeszow University, Rzeszów, Poland

**Keywords:** nurses, physical activity, metabolic disorders, accelerometer, public health

## Abstract

Numerous studies demonstrate a relationship between physical activity and the development of non-communicable diseases. Nurses play a crucial role in the healthcare system, and their demanding work can have an impact on their health. The objective of this cross-sectional study was to assess physical activity in relation to factors predisposing to the occurrence of specific metabolic disorders among Polish nurses. The measurements included physical activity level using ActiGraph GT3X, body weight composition using Tanita MC-980, body mass index, waist circumference, blood pressure using Welch Allyn 4200B, lipid profile, and fasting blood glucose using CardioChek PA. The results indicate that nearly one-third (31.75%) of the total sample of studied nurses do not meet the criteria for the minimum amount of physical activity of at least moderate intensity. Furthermore, over half of the surveyed nurses (55.5%) were classified as overweight or obese based on BMI, and almost half (42.86%) had abdominal obesity. The regression model, employing linear regression, revealed that factors predisposing to selected metabolic disorders were age, engaging in multiple jobs, and the number of steps per day. There is a pressing need to implement comprehensive and supportive initiatives to improve the overall health condition of nurses in Poland through increased physical activity. Activating and supporting this professional group is an investment that benefits not only the nurses themselves but also the healthcare system and the entire nation.

## 1 Introduction

The health benefits of an appropriate level of physical activity (PA) are evident and indisputable (1, 2). Recent trends in assessing physical activity involve the development and implementation of national guidelines for physical activity across all age groups, routine surveillance and monitoring of physical activity, policy implementation, and the use of tools such as the Global Physical Activity Questionnaire (GPAQ) and accelerometers for nationwide population monitoring of physical activity in both adults and children (3, 4). Studies have also investigated temporal patterns in physical activity levels among different age groups (5, 6), advancements and emerging directions in physical activity surveillance (7), and recent tendencies in adherence to physical activity guidelines (8–10). Moreover, research has examined the close association between physical activity and the prevalence of non-communicable diseases (11). Despite numerous studies highlighting the benefits of physical activity, many adults and children fail to meet the recommended levels of physical activity (4, 12). Nurses possess knowledge about healthy behaviors and recognize the importance of physical activity for maintaining health (13, 14). However, research indicates that compared to other occupational groups, nurses often report poorer health and engage in unhealthy behaviors, such as physical inactivity, unhealthy eating habits, and smoking (15–17). These factors, combined with shift work, heavy workloads, and high levels of stress inherent in nursing, contribute to an increased risk of developing various diseases within this profession and a decline in the quality of patient care (17–19).

The demanding work environment of nurses requires not only comprehensive preparation but also mental resilience and physical fitness (20, 21). Nursing tasks must be performed efficiently and accurately, thus placing high demands on the physical fitness of nurses as it directly impacts the provision of proper patient care (21–24). Nurses experience a higher number of stressful situations compared to other professions, including other healthcare sector employees (25, 26). Regular physical activity improves general physical well-being and is a strategy to reduce work-related tension, stress and protect against professional burnout (27–29).

Nurses constitute the largest occupational group within the healthcare sector (30) and conducting research on this topic offers an excellent opportunity to emphasize the significance of physical activity not only for their personal health but also for the effective functioning of healthcare systems. The objective of this study was to assess physical activity in relation to factors predisposing to the occurrence of specific metabolic disorders.

## 2 Materials and methods

The study was conducted in 2022 among 126 professionally active nurses working at the clinical hospital in the Subcarpathian region.

### 2.1 Procedures

The project was approved by the hospital manager and head nurse. In collaboration with hospital authorities, information regarding the dates, hours, and extent of the planned examinations was provided to all nurses working in individual hospital wards. Nurses who wished to participate in the study signed up on the prepared list and selected a convenient date. All measurements (including body composition analysis, glucose level, and lipid profile) were conducted in the morning after a minimum fasting period of 8 h.

Accelerometers were assigned individual codes for each participant, taking into account their age, weight, and height. They were immediately worn by the participants following the measurements. The inclusion criteria consisted of professionally active nurses without any recent symptoms of infection within the past 2 weeks, no known health issues, and a willingness to participate in the project, which included wearing the accelerometer for seven consecutive days.

Initially, 154 nurses expressed interest in participating in the physical activity measurement using the accelerometer. However, within 2 days, 7 of them reported being unable to continue due to external circumstances. An additional 11 nurses were excluded from the analysis as it was observed during the data review that the device was not worn according to the established criteria. Ultimately, data from 126 accelerometers were included in the statistical analysis.

### 2.2 Blood pressure

Blood pressure was measured three times in the sitting position, after a 10-min rest, following the recommendations of the European Society of Hypertension (31). The measurements were taken using a standardized Welch Allyn 4200B apparatus (Aston Abbotts, UK). The average of the three measurements was calculated for each participant.

The accepted criteria for blood pressure were as follows:

- normal blood pressure ranged from 120–129 mmHg systolic and 80–84 mmHg diastolic;
- normal high pressure ranged from 130–139 mmHg systolic and 85–89 mmHg diastolic;
- grade 1 hypertension ranged from 140–159 mmHg systolic and 90–99 mmHg diastolic;
- grade 2 hypertension ranged from 160–179 mmHg systolic and 100–109 mmHg diastolic;
- grade 3 hypertension was defined as a systolic blood pressure of ≥180 mmHg and/or a diastolic blood pressure of ≥110 mmHg (31).

### 2.3 Anthropometry

Body height was measured to an accuracy of 0.1 cm using a Seca 213 stadiometer in a vertical position, with the participant barefoot (Seca, Hamburg, Germany). Bodyweight was assessed in the early morning after at least 8 h of overnight fasting, in light clothing, in an upright position, with an accuracy of 0.1 kg, using food-to-food bioelectric impedance analysis with a Tanita MC-980 PLUS MA (Tanita, Tokyo, Japan) (32).

Body Mass Index (BMI) was calculated as the participant’s body weight in kilograms divided by their height in meters squared, based on accepted standards:

17–18.49 = underweight,

18.5–24.99 = normal body weight,

25–29.99 = overweight,

30–34.99 = 1st degree obesity.

35–39.99 = 2nd degree obesity,

>40 = 3rd degree obesity (33).

Waist and hip girths were measured with steel anthropometric tape in accordance with the International Society of the Advancement of Kinanthropometry (ISAK) guidelines by an ISAK Level 3 – Instructor anthropometrist.

Waist-to-hip ratio (WHR) was calculated as the participant’s waist circumference divided by their hip circumference. The following ranges were adopted for the value of the WHR index:

For men:

<0.96 WHR within normal limits.

≥0.96 Abdominal obesity, increased risk of metabolic diseases.

For women:

<0.83 WHR within normal limits.

≥0.83 Abdominal obesity, increased risk of metabolic diseases (34).

### 2.4 Blood biochemical measurements

Lipid profile and fasting glucose were assessed using the CardioChek PA (CCPA, PTS Diagnostics, Whitestown, USA) analyzer according to the manufacturer’s instructions. A finger prick capillary blood sample was collected by a registered nurse while observing all rules of asepsis and antisepsis. Blood samples were collected in the morning after an 8-h fast. Total cholesterol (TC), low-density lipoprotein (LDL) cholesterol, high-density lipoprotein (HDL) cholesterol, triglycerides (TG), and glucose levels were measured. Quality control testing of the device was done prior to data collection with a Multi-Chemistry Controls set. The CCPA device was checked each time before starting by conducting an Internal Quality Control using a control strip recommended by the manufacturer.

In accordance with the guidelines of the Polish Cardiological Society, the following lipid profile criteria have been adopted:

- TC 150–190 mg/dL;
- LDL cholesterol less than 115 mg/dL;
- HDL cholesterol above 40 mg/dL for men and over 48 mg/dL for women;
- TG below 150 mg/dL.

Lipid disorders criteria:

- Hypercholesterolemia was diagnosed when TC was ≥190 mg/dL or LDL > 115 mg/dL;
- Atherogenic dyslipidaemia was determined when TG was ≥150 mg/dL, HDL < 40 mg/dL in males and < 48 mg/dL in females, and elevated LDL fraction (>130 mg/dL);
- Hypertriglyceridemia was determined when TG was >150 mg/dL with normal LDL levels (<115 mg/dL) and severe hypertriglyceridemia – TG ≥ 800 mg/dL;
- Dyslipidaemia was defined as the presence of abnormal concentrations in any component of the lipid profile (35–37).

Fasting glucose criteria:

- less than 70 mg/dL – hypoglycemia;
- 70 to 99 mg/dL – normal glucose level;
- 100 to 125 mg/dL – elevated glucose levels – pre-diabetes;
- ≥ 126 mg/dL at least two measurements – diabetes mellitus (35–37).

### 2.5 Physical activity

Physical activity parameters were measured using the ActiGraph GT3X-BT triaxial accelerometer (ActiGraph, Pensacola, Florida, USA). Accelerometers have been widely used to assess physical activity and are considered a valid and reliable measure of physical activity in many populations, including adults (38, 39).

The accelerometer was worn on the subject’s right hip. Subjects were instructed to wear the monitor for seven consecutive days, 24 h a day, and remove it only for water-related activities (e.g., showering or swimming). The sleep-period time was separated from the 24-h activity with the Sadeh algorithm (40). Non-wear time was defined as a period of at least 60 consecutive minutes with zero counts per minute (cpm). The epoch duration was set to 60 s. A minimum wear time of ≥600 min per day was considered a valid day for the analysis. At least 4 days (including at least three valid weekdays and one valid weekend day) were used as the criteria for a valid seven-day period of accumulated data.

After the recording period ended, the accelerometers were connected to a computer via a mini-USB for data transfer. Data were analyzed using ActiLife software (ActiGraph, software v.6.13, Pensacola, FL, USA). For each participant, time (minutes per week and minutes per day) spent in sedentary, light, moderate, vigorous, moderate-to-vigorous physical activity (MVPA), and the average daily and weekly step counts were calculated. The cut-off points for sedentary (0–99 cpm), light physical activity (100–1,951 cpm), moderate (1,952–5,724 cpm), vigorous (≥5,725 cpm), and MVPA (≥1,952 cpm from all valid days) were applied (41).

In the present study, the World Health Organization’s (WHO) global recommendations on physical activity for adults were adopted. The cut-off points for meeting the guidelines were set up as 150 min of moderate-intensity aerobic physical activity throughout the week or at least 75 min of vigorous-intensity aerobic physical activity throughout the week or an equivalent combination of moderate- and vigorous-intensity activity (3).

### 2.6 Questionnaire

The questionnaire developed for this study was prepared in a paper format, which included an envelope for sealing the completed questionnaires to ensure the confidentiality of the responses. The survey consisted of questions regarding the participants’ sociodemographic data (such as age, sex, place of residence, and level of education), work-related questions (such as type of work, work schedule, number of full-time jobs, and years of experience), participation in preventive examinations, smoking habits, and the prevalence of chronic diseases among the surveyed nurses (Appendix 1 – questionnaire template).

### 2.7 Applied statistical methods

The analysis was performed using the R program, version 4.2.2 (42). The analysis of quantitative variables was performed by calculating the mean, standard deviation, median, and quartiles. The analysis of qualitative variables was performed by calculating the number and percentage of occurrences of each value.

Multivariate analysis was conducted to assess the influence of multiple variables on the occurrence of individual metabolic disorders. This analysis employed the logistic regression method, which was adjusted to age, shift work, working more than one full-time job, smoking, and chronic diseases.

The results are presented as Adjusted Odds Ratio (AOR) parameter values along with 95% confidence intervals.

In this analysis, a significance level of 0.05 was adopted. Therefore, any *p*-values below 0.05 were considered indicative of significant relationships.

## 3 Results

### 3.1 Socio-demographic characteristics of the study group

Totally, 126 nurses (women) were included to final analysis. The average age of the respondents was approximately 46.5 years (SD ± 9.95), median (quartiles), 50 (39–54), range 23–65. Detailed characteristics of the study group are presented in Table 1.

**Table 1:** General characteristics of the study group.

Variable		Total (*n* = 126) *n* (%)
Place of residence*	City	63 (50.0)
	Village	63 (50.0)
Type of work*	Staff management/administration	22 (17.5)
	Hospital ward	104 (82.5)
Work system*	Shift work and night duty (12 h)	64 (50.8)
	One shift work (8 h)	62 (49.2)
More than one job*	No	102 (80.9)
	Yes	24 (19.1)
Education*	Basic nursing education	20 (15.9)
	Bachelor	39 (30.9)
	Master’s degree	67 (53.2)
Participation in preventive examinations other than obligatory*	No	91 (72.2)
	Yes	35 (27.8)
Cigarettes smoking*	No	110 (87.3)
	Yes	16 (12.7)
Chronic diseases*	No	78 (61.9)
	Yes	48 (38.1)
	Anthropometric characteristics	
BMI* [kg/m^2^]	Normal body mass	56 (44.4)
	Overweight	40 (31.8)
	Class I obesity	19 (15.1)
	Class II obesity	9 (7.1)
	Class III obesity	2 (1.6)
WHR*	Normal	72 (57.1)
	Abdominal obesity. Increased risk of metabolic diseases	54 (42.9)
HDL*	Normal	120 (95.2)
	To low	6 (4.8)
TG*	Normal	103 (81.8)
	Elevated	23 (18.2)
LDL*	Normal	82 (65.1)
	Elevated	44 (34.9)
TC*	Normal	82 (65.1)
	Elevated	44 (34.9)
Lipid disorders*	No	67 (53.2)
	Yes	59 (46.8)
BP* [mmHg]	Normal	77 (61.1)
	Elevated	21 (16.7)
	High blood pressure Stage 1	26 (20.6)
	High blood pressure Stage 2	2 (1.6)
FG* [mg/dL]	Normal	80 (63.4)
	Elevated	44 (34.9)
	Abnormal glucose – suspicion of diabetes	2 (1.6)

* Data presented as: *, n (%); SD, standard deviation; TC, total cholesterol; HDL, high-density lipoprotein; LDL, low-density lipoprotein; TG, triglycerides; BP, Blood pressure; BMI, Body mass index; WHR, Waist Hip Ratio; FG, Fasting glucose.

### 3.2 Quantity and quality of physical activity undertaken by the surveyed nurses

Descriptive statistics of accelerometer data, amount, and type of physical activity per day and week are shown in Table 2.

**Table 2:** Parameters of physical activity in the study group (*n* = 126).

Parameter	M	SD	Me	Min	Max	Q1	Q3
Sedentary [min per week – sleep]	3976.7	1581.1	3802.6	1282.0	13635.7	3074.7	4373.4
Sedentary [min per day – sleep]	594.0	134.2	574.9	305.7	1050.5	497.82	685.4
Light activities [min per week]	2657.3	805.0	2679.0	180.5	4456.0	2112.5	3263.5
Light activities [min per day]	397.7	112.3	398.0	25.7	674.7	330.1	479.2
Moderate activities [min per week]	225.5	136.8	194.6	11	672	127.5	287
Moderate activities [min per day]	33.8	20.4	28.6	1.8	101.4	19.42	41.5
Vigorous activities [min per week]	6.4	19.9	0	0	149	0	1.9
Vigorous activities [min per day]	0.9	3.0	0	0	22.5	0	0.3
Total MVPA [min per week]	232	141.4	202.5	11	720	127.5	297.2
Average MVPA [min per day]	31.9	19.8	28.5	1.4	102.9	18	41.6
Steps counts per week	64982.7	22658.0	64681.5	8,025	122,190	51232.5	79185.7
Steps counts per day	9732.0	3282.2	9773.5	1331.5	18,502	7692.0	11758.3

M, arithmetic mean; SD, standard deviation; Me, Median; Min, Minimum; Max, Maximum; Q1, lower quartile; Q3, upper quartile.

The results showed the amount and type of physical activity undertaken by nurses, as well as the number of steps taken in relation to the recommended standards (Table 3).

**Table 3:** Level of physical activity and number of steps in relation to the recommendation.

Parameter			Total (*n* = 126) n (%)
* Physical activity per week (in relation to the recommendation)	Meet criteria	No	40 (31.7)
		Yes	86 (68.3)
Steps per day	Active lifestyle – meets or exceeds the recommended number of steps per day (≥10,000)		61 (48.4)
	Somewhat active lifestyle – meets the recommended number of steps per day (7,500–9,999)		36 (28.6)
	Low lifestyle – does not meet the recommended number of steps per day (7,499–5,000)		21 (16.7)
	Sedentary lifestyle – does not meet the requirements for the recommended number of steps per day (<5,000)		8 (6.3)

* 150–300 min of moderate-intensity or 75–150 min of vigorous-intensity per week.

### 3.3 Activity variables influencing the occurrence of metabolic disorders

Multivariate logistic regression analysis, adjusted to age, shift work and night duty, more than one job, cigarettes smoking and chronic diseases, showed no significant relationships (*p* > 0.05) between physical activity per week and occurrence selected metabolic disorders (Table 4).

**Table 4:** Physical activity per week and the occurrence of metabolic disorders in the study group.

Physical activity per week	Overweight	Obesity	Abdominal obesity	Lipid disorders**	Elevated blood pressure	Elevated glucose levels
Meet criteria	1	1	1	1	1	1
Do not meet criteria	AOR = 1.039 (0.472–2.29) *p* = 0.924	AOR = 0.984 (0.382–2.538) *p* = 0.974	AOR = 1.58 (0.673–3.707) *p* = 0.293	AOR = 1.415 (0.638–3.136) *p* = 0.393	AOR = 0.625 (0.266–1.466) *p* = 0.28	AOR = 0.632 (0.271–1.471) *p* = 0.287

*Statistically significant relationship (*p* < 0.05); AOR (Adjusted Odds Ratio) = odds ratio (OR), adjusted to age, shift work, working more than one full-time job, smoking, and chronic diseases, **lipid disorders – at least one of the parameters is disturbed.

Multivariate logistic regression analysis, adjusted to age, shift work and night duty, more than one job, cigarettes smoking and chronic diseases, showed that an inactive lifestyle (7,499–5,000 steps per day) increases the risk of lipid disorders on average 5.712 times (AOR = 5.712) compared to a very active lifestyle (≥10,000) (Table 5).

**Table 5:** Steps per day and the occurrence of metabolic disorders in the study group.

Steps per day	Overweight	Obesity	Abdominal obesity	Lipid disorders**	Elevated blood pressure	Elevated glucose levels
≥10,000	1	1	1	1	1	1
7,500–9,999	AOR = 0.833 (0.35–1.985) *p* = 0.68	AOR = 1.517 (0.542–4.241) *p* = 0.427	AOR = 1.083 (0.415–2.83) *p* = 0.871	AOR = 0.534 (0.218–1.306) *p* = 0.169	AOR = 0.836 (0.326–2.147) *p* = 0.711	AOR = 1.521 (0.609–3.799) *p* = 0.369
7,499–5,000	AOR = 1.25 (0.434–3.601) *p* = 0.679	AOR = 1.177 (0.332–4.176) *p* = 0.801	AOR = 2.251 (0.675–7.502) *p* = 0.187	AOR = 5.712 (1.497–21.798) *p* = 0.011*	AOR = 1.166 (0.387–3.514) *p* = 0.785	AOR = 0.43 (0.12–1.538) *p* = 0.194
<5,000	AOR = 7.296 (0.767–69.433) *p* = 0.084	AOR = 0.336 (0.03–3.797) *p* = 0.378	AOR = 0.373 (0.055–2.524) *p* = 0.312	AOR = 0.601 (0.115–3.146) *p* = 0.547	AOR = 0.305 (0.046–2.005) *p* = 0.216	AOR = 1.622 (0.317–8.298) *p* = 0.561

* Statistically significant relationship (*p* < 0.05); AOR (Adjusted Odds Ratio) = odds ratio (OR), adjusted to age, shift work, working more than one full-time job, smoking, and chronic diseases, ** lipid disorders – at least one of the parameters is disturbed.

Multivariate logistic regression analysis, adjusted to age, shift work and night duty, more than one job, cigarettes smoking and chronic diseases, showed no significant relationships (*p* > 0.05) between sedentary (h/day) and occurrence selected metabolic disorders (Table 6).

**Table 6:** Sedentary and the occurrence of metabolic disorders in the study group.

Sedentary [h/day]	Overweight	Obesity	Abdominal obesity	Lipid disorders**	Elevated blood pressure	Elevated glucose levels
[h/day]	AOR = 1.143 (0.954–1.368) *p* = 0.146	AOR = 1.007 (0.829–1.223) *p* = 0.942	AOR = 0,892 (0.736–1.081) *p* = 0.243	AOR = 1.065 (0.894–1.269) *p* = 0.48	AOR = 1.027 (0.859–1.227) *p* = 0.77	AOR = 1.007 (0.84–1.207) *p* = 0.941

* Statistically significant relationship (*p* < 0.05); AOR (Adjusted Odds Ratio) = odds ratio (OR), adjusted to age, shift work, working more than one full-time job, smoking, and chronic diseases, ** lipid disorders – at least one of the parameters is disturbed.

## 4 Discussion

The results demonstrated the impact of objectively measured physical activity, number of steps, sitting time, age, work pattern, and additional employment on the prevalence of obesity, including overweight, abdominal obesity, lipid disorders, elevated blood pressure, and elevated blood glucose levels. Increasing evidence suggests the importance of physical activity for better health and the prevention of non-communicable diseases (43–47). This study is one of the first to objectively assess physical activity among nurses in Poland. The aim was to evaluate the physical activity of nurses in relation to factors that predispose them to metabolic disorders. This cross-sectional study found that almost one third (31.75%) of the nurses studied did not meet the criteria for a minimum amount of physical activity of at least moderate intensity. More than half of the nurses surveyed (55.56%) were overweight or obese according to BMI and almost half (42.86%) had abdominal obesity.

Overweight and obesity have been a recognized health problem for many years, affecting not only Poland but also other countries such as Great Britain, Australia, the United States, and New Zealand (48–52). Other studies have also shown that the prevalence of overweight and obesity among nurses is more frequent than among the general population and other employees in the health sector (50–53). For example, Kayaroganam et al. found that one-fifth of the nurses surveyed were overweight, more than a third had abdominal obesity, and more than half were obese, including women over 40 (54).

In our study, lipid disorders were present in 46.83% of the patients, and 38.89% of the nurses had elevated blood pressure. The specificities of the nurses’ work, such as irregular lifestyles, unhealthy eating habits, shift work, and disturbances of the natural rhythm of sleep and rest, may predispose them to many disorders, such as dyslipidemia, hypertension, and elevated glucose levels. Consequently, these factors may lead to the development of cardiovascular diseases, which is a global problem that accounts for the majority of deaths, especially in developed countries. The results of the study conducted by the Cardiovascular Nurses Associates indicate the prevalence of the above relationships among nurses (55). Researchers from South India have also shown that more than one-third of nurses had hypercholesterolemia (34.3%) and elevated LDL (41.9%), and two-thirds had low HDL (65.3%) (54). Hypertension is another global problem that affects more and more people and leads to morbidity and premature death (56). In the Manakali study, 52% of nurses had hypertension, which is significantly higher than the reported prevalence of hypertension among nurses in South Africa (20%), Brazil (32%), and healthcare professionals in Nigeria (20.1%). This result is also higher than the documented prevalence of hypertension in the general adult population of South Africa (57, 58). Based on the findings of our study, 36.51% participants had elevated glucose levels, which is higher than in the Kayarogana study (11.5%) (54). However, a study conducted by Miller et al. on health risk factors of nurses showed that 26% of nurses participating in the measurements were unaware of their diabetes (59). Other studies indicate that elevated glucose levels among nurses may also result from stress experienced during their daily work (60, 61).

The results in relation to physical activity showed that the nurses participated mainly in light and moderate intensity physical activity and to a lesser extent in vigorous activities. When analyzing the results of the entire study group, the average MVPA was 232 min per week, indicating that the criteria for the recommended amount of physical activity for an adult per week have been met. However, based on the results, 31.75% of nurses do not meet the minimum requirements for a minimum amount of physical activity of at least moderate intensity. The result obtained is higher compared to the data from the Ministry of Sport and Tourism report, which showed that approximately 80% of the Polish society does not engage in moderate or intense physical activity, and about 23% declare moderate activity, mainly related to the need to move (62). However, considering the nature of the nursing profession and the need to constantly move while caring for patients (63), the result obtained cannot be considered fully satisfactory. Ottawa Canadian researchers conducted an objective evaluation of physical activity among 364 nurses using accelerometers. Their results showed that only a few nurses (23%) met the WHO physical activity guidelines (≥150 min/week in bouts of ≥10 min) (64). According to a systematic review by Chappel et al. nurses are mostly engaged in light-intensity physical activity (65). Other researchers have presented similar results in this regard. According to a survey conducted by Tucker et al. among 3,132 nurses, only 50% of them reported that they met the guidelines for physical activity (66). In a cross-sectional study of 325 British nurses by Blake, Malik, Mo and Pisano, they found that less than half (45.98%) met the government guidelines for physical activity (67).

In our study, nurses accumulated an average of 232 min per week of moderate to vigorous intensity physical activity. On the other hand, Canadian nurses achieved higher results in this respect, with an average of 288 min per week (64). The number of steps per day is a simple measure of physical activity, which is now easily monitored thanks to various electronic devices that are increasingly available and popular (43, 44). Our step count results showed that the average number of steps per day was 9,732. This result is in line with the majority of recommendations on the optimal number of steps per day for good health (43). These findings suggest that the nurses who participated in our study are more active in this regard than the general population of Poland, where the average person takes about 5,000 steps per day (68). Data indicate that the highest number of steps per day are taken by people in Switzerland (10,400 for men and 8,900 for women), followed by Belgians (9,500), Japanese (7,200) and then Americans (6,500) (69). When assessing the number of steps per day among nurses, many researchers focus on measuring the number of steps during a 12 and / or 8-h shift (63, 70, 71). Croteau et al. showed that nurses take more steps on working days (10,398) compared to non-working days (7,036) (72). There are several recommendations in this regard (73), but the goal of achieving 10,000 steps per day for healthy individuals has been widely promoted and advocated by the WHO and various physical activity campaigns (43, 74).

The multivariate logistic regression analysis, adjusted to age, shift work and night duty, more than one job, cigarettes smoking and chronic diseases, showed that an inactive lifestyle (7,499–5,000 steps per day) increases the risk of lipid disorders on average 5.712 times (AOR = 5.712) compared to a very active lifestyle (≥10,000). No significant relationships between physical activity per week, between sedentary (h/day) and occurrence selected metabolic disorders (*p* > 0.05). A meta-analysis of 153,030 nurses from 35 studies demonstrated that shift work, age, night shift, sex, marriage, health status, number of hours worked per week and stress level are positively correlated with overweight and obesity among nurses (75). Researchers from Ghana also found that age, sex, and marital status influence the level of obesity and overweight among nurses, with older nurses being more likely to be obese than younger ones (70, 76). In Poland, the majority of nurses currently working are 46–60 years old (77), which underscores the importance of paying attention to improving the health condition of nurses in the context of an increased risk of developing metabolic disorders and even mortality (78). A meta-analysis conducted by Sheng et al. shows that taking 9,500 steps per day reduces the risk of cardiovascular events by approximately 35% compared to 3,500 steps per day (78). The authors of another meta-analysis showed that among people over 60 years of age, taking 6–9 thousand steps per day reduces cardiovascular risk by up to 40–50% compared to taking approximately 2000 steps per day (79). According to Sheng, taking 8,959 steps a day reduces the risk of death by 40.36% compared to taking 4,183 steps a day (78). Garduno et al. showed that every additional 2000 steps per day reduces the risk of diabetes by 12% (80). The benefits of taking the recommended number of steps include weight loss and all the consequences of being overweight and obesity. Additionally, physical activity has a positive effect on stress reduction, which is often present in the work of nurses (81). The results of a meta-analysis published in The Lancet indicate that taking 6–9,500 steps a day is optimal, noting that the more steps taken, the better (47).

The shortage of nursing staff is a problem currently faced by many countries, including Poland (30). The average age of a Polish nurse is currently older than 53 years, meaning that the majority of currently working nurses have reached or will soon reach retirement age. This predisposes them to poor health and absenteeism, which is crucial not only for themselves but also for ensuring high quality healthcare (77). Analyses of millions of patient records in the United States and Canada indicate a direct relationship between care satisfaction and the number of adverse events and the health condition of nursing staff (82). Nurses are the largest occupational group among health professionals and reach a large proportion of the population, making them an integral part of the health workforce (30). According to Fie et al., nurses who have a positive attitude toward physical activity and are physically active are more likely to promote physical activity among their patients compared to inactive nurses (83, 84). A very positive solution is the national campaign organized by the American Nurses Association, which supports and promotes pro-health behaviors among nurses (85).

### 4.1 Strengths, limitations, and future research

To our knowledge, this is the first objective study in Poland that measures physical activity among nurses using certified devices. Most of the research in this area is subjective survey research. The strength of our study is the objective, not the declared assessment of physical activity and the performance of all measurements using standardized high-quality devices. However, several potential study limitations should be considered when interpreting the results. Firstly, since the study is cross-sectional, causality and temporality issues cannot be considered. Second, only women participated in the study, since men make up less than 2% of all nurses, so it was difficult to include them in the study group when randomly selected. Third, the study had a limited geographic scope and should be expanded to more medical facilities in other regions. Fourth, due to limited availability of devices at our disposal (high price), we recruited only some of the nurses. It is possible that those who did not participate in our measurements were more physically active. Future research should include male participation, a larger group of nurses, and consideration of other regions of the country. Our work is a preliminary assessment and provides the basis for implementing multifaceted and supportive activities to improve the general health condition of nurses in Poland by increasing physical activity.

Future research could include an audit to objectively assess infrastructure and opportunities to increase physical activity.

## 5 Conclusion

The results of this study indicate that the level of physical activity and the presence of factors that contribute to the development of metabolic diseases can have significant implications for the health of nurses, as well as the effectiveness of their work and the quality of patient care. Providing care to those who care for us is necessary, given the requirements imposed by the nurse’s profession and the patient’s right to receive the highest level of care, regardless of age and health condition. Therefore, appropriate interventions should be designed and implemented not only to increase and maintain the level of physical activity of nurses, but also to eliminate barriers that make it difficult to carry out health activities. The activation and support of this professional group is an investment that benefits not only the nurses themselves, but also the health care system and the entire nation.

## Data availability statement

The raw data supporting the conclusions of this article will be made available by the authors, without undue reservation.

## Ethics statement

The study involving humans was approved by the Bioethics Committee of the University of Rzeszów (No. 2022/088 of 05/10/22) and was conducted under the ethical standards stated in the Declaration of Helsinki. The participants provided their written informed consent to participate in this study.

## Author contributions

AB: Conceptualization, Data curation, Formal analysis, Investigation, Methodology, Writing – original draft, Writing – review & editing. PM: Data curation, Methodology, Writing – review & editing. JW: Data curation, Investigation, Methodology, Writing – review & editing. EŁ: Data curation, Writing – review & editing. ŁO: Funding acquisition, Writing – review & editing. OA: Funding acquisition, Writing – review & editing. AM-R: Writing – review & editing. AM: Writing – review & editing.
