# Supplementary material for: Levels of actigraphy-derived physical activity among Polish nurses: factors associated with the prevalence of selected metabolic disorders
Source: Front Public Health. 2023 Dec 29;11:1300662. doi: 10.3389/fpubh.2023.1300662 (PMC10787602; doi:10.3389/fpubh.2023.1300662)
Supplement: Supplementary file 1 [file Data_Sheet_1.pdf]

☐☐☐☐☐

Questionnaire

We kindly ask you to complete the survey, as a supplement to the study on the assessment of physical activity among nurses.

Please mark an "X" next to the selected answer that best applies to your situation or fill in the response in the designated space.

1. Age ..........
2. Sex: ☐ female ☐ male
3. Place of residence: ☐ city ☐ village
4. Type of work
   - ❑ Staff management/administration
   - ❑ Hospital ward
5. Work system
   - ❑ Shift work and night duty (12h)
   - ❑ One shift work (8h)
6. Do you work more than one job?
   - ❑ No
   - ❑ Yes
7. Level of education
   - ❑ Basic nursing education
   - ❑ Bachelor
   - ❑ Master degree
8. Do you participate in preventive examinations other than obligatory?
   - ❑ No
   - ❑ Yes
9. Do you smoke?
   - ❑ No
   - ❑ Yes
10. Do you have any chronic diseases?
    - ❑ No
    - ❑ Yes
